# Impact of a 2-yr anti-TNF treatment on the quality of life in JIA patients: a parent's/patient’s view

**DOI:** 10.1186/1546-0096-9-S1-P125

**Published:** 2011-09-14

**Authors:** K Diafa, P Pratsidou-Gertsi, M Trachana, G Pardalos, F Kanakoudi-Tsakalidou

**Affiliations:** 1 Pediatric Immunology and Rheumatology Referral Center, 1st Department of Pediatrics, Aristotle University, Ippokration General Hospital, Thessaloniki, Greece

## Background

The use of biologic factors in the management of Juvenile Idiopathic Arthritis (JIA) has changed dramatically the course and outcome of the disease. They control the disease symptoms and hinder the joint damage which may provoke major functional limitations, thus affecting the patients’ Health Related Quality of Life (HRQoL).

## Aim

To evaluate changes in the HRQoL variables in patients with JIA, under anti-TNF treatment.

## Methods

49 patients with refractory to conventional therapy JIA, aged 2-18 years, were studied. They were receiving either Etanercept or Adalimumab for the first time or they were switchers from one anti-TNF to the other one (10/49). All patients were clinically and laboratory assessed pre-treatment and thereafter every 6 months, until the completion of 2 years. At the same time-points, the following variables were collected: the Childhood Health Assessment Questionnaire (CHAQ) Disability Index (DI), the patient’s/parent’s global assessment of pain and wellbeing (P/PVAS) and the physician’s global assessment (MDVAS) through the visual analogue scale (VAS).

## Results

During anti-TNF treatment, all the above mentioned core set variables P/PVAS showed a significant improvement (p<0.05). Similarly, the improvement of MDVAS was positively correlated with the improvement of CHAQ DI (rsq=0.43), of VAS pain (rsq=0.66) and of VAS wellbeing (rsq=0.64). No correlation between VAS wellbeing and JIA duration was found. Pain was the only independent factor related with a poor score of wellbeing.

## Conclusion

This study shows that all variables associated with HRQoL in patients with JIA can be substantially improved after 2 years of anti-TNF treatment.

